# From Tradition to the Future: Analyzing the Factors Shaping Consumer Acceptance of Cultured Meat Using Structural Equation Modeling

**DOI:** 10.1002/fsn3.70435

**Published:** 2025-06-13

**Authors:** Feray Gençer Bingöl, Duygu Ağagündüz

**Affiliations:** ^1^ Department of Nutrition and Dietetics, Faculty of Health Science Burdur Mehmet Akif Ersoy University Burdur Türkiye; ^2^ Department of Nutrition and Dietetics, Faculty of Health Science Gazi University Ankara Türkiye

**Keywords:** alternative protein sources, animal welfare, cultured meat, food neophobia, sustainable nutrition

## Abstract

Sustainable nutrition promotes reducing meat consumption due to environmental concerns. In this context, alternative protein sources, such as cultured meat, are discussed as an option to traditional meat consumption. This cross sectional study examined expectations and concerns regarding cultured meat. Data were collected via an online questionnaire, assessing acceptance, expectations, concerns, sustainable nutrition, food neophobia, and animal welfare. A total of 504 young adults (18–40 years) participated. Among them, 61.1% had heard of cultured meat, and 33.5% were open to consuming it. Acceptance decreased with age (β: −0.026; *p* < 0.001) but increased with higher education (β: 0.146; *p* < 0.05). Structural equation modeling showed that expectations positively influenced acceptance (β: 0.207; *p* < 0.001), whereas concerns had no direct effect. Food neophobia reduced expectations but did not directly affect acceptance. Greater awareness of sustainable nutrition (β: 0.169; *p* < 0.001) and animal welfare (β: 0.154; *p* < 0.05) increased concerns about cultured meat but did not impact acceptance. Strengthening consumer expectations has the potential to enhance cultured meat acceptance. However, this study contributes to previous literature by highlighting how these expectations interact with specific psychological and ethical factors such as sustainable nutrition, food neophobia, and animal welfare. The results underscore the importance of transparent information strategies that address not only concerns but also the motivational drivers influencing consumer decisions in the context of cultured meat.

## Graphical abstract

Acceptance of cultured meat decreased with increasing age, but increased with higher educational status. Structural equation modeling revealed that expectations had a positive effect on the acceptance of cultured meat, whereas concerns had no direct effect. Although food neophobia reduced expectations, it did not have a direct impact on acceptance. Awareness of sustainable nutrition and animal welfare increased concerns regarding cultured meat but did not influence acceptance of cultured meat.

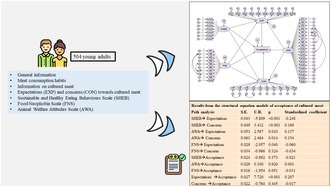

## 1 Introduction

The increase in the world population accelerates the depletion of food and natural resources, highlighting the need for sustainable strategies to address potential food shortages (Duro et al. 2020). Meat consumption is one of the most discussed food groups in terms of sustainability. Increased meat consumption not only leads to a decrease in limited resources but also increases greenhouse gas emissions. In addition, it creates a heavy environmental burden by increasing water, energy, and land use (Parlasca and Qaim 2022).

Regarding meat consumption, the Türkiye Nutrition Guideline 2022 recommends a total daily consumption of 60 g of red meat and chicken for adults aged 19 and over (Republic of Türkiye Ministry of Health 2022). According to the results of the Türkiye Nutrition and Health Survey 2017, the daily red meat consumption amount is 39.5 g, and the chicken meat consumption amount is 28.2 g (Republic of Türkiye Ministry of Health 2019). According to a joint report by the Organization for Economic Cooperation and Development and the Food and Agriculture Organization, whereas the world's meat consumption per capita was 42.7 kg/year in 2020, this amount is expected to increase to 43.7 kg/year in 2030 (OECD, Food, and of the United Nations 2021). The EAT‐Lancet Commission defined an average daily consumption of 14 g of red meat (0–28 g) and 29 g of poultry (0–58 g) as reference values in the dietary model it presented for sustainable food systems in 2019 (Willett et al. 2019). These results indicate that to attain sustainable and nutritionally balanced diets and prevent additional rises in meat consumption, there is a need to minimize meat consumption globally. Meat reduction strategies can help reduce the environmental burden of traditional meat production. Meat reduction strategies include controlling portion sizes, raising awareness about environmental impacts and animal welfare, communicating potential adverse health effects, and choosing alternative protein sources to meat (Harguess et al. 2020; Mustapa et al. 2025; van den Berg et al. 2022).

Legumes, plant‐based alternative proteins, algae, insects, and cultured meat are among the alternative protein sources. The acceptability of alternative protein sources is seen to be lower compared to meat (Onwezen et al. 2021). Therefore, research focuses on the acceptability of alternative protein sources by consumers (Kasza et al. 2023; Melendrez Ruiz et al. 2019; Moussaoui et al. 2023). Although the acceptability of alternative protein sources such as legumes and plant‐based proteins appears to be relatively high, consumer attitudes toward insects and cultured meat remain uncertain and hesitant (Onwezen et al. 2021). In this context, cultured meat has a special importance as a potential transition product for consumers, as it is distinguished from other alternative protein sources by its closeness to traditional meat in taste, texture and nutritional content. In addition, cultured meat has more uncertainty in terms of consumer acceptance compared to some other alternative proteins, which requires prioritization in research (Fraeye et al. 2020).

Cultured meat has been produced in a laboratory environment as a novel alternative protein source through the application of advanced biotechnology. Globally, cultured meat is also known by various names, such as “cultivated meat,” “in vitro meat,” “lab‐grown meat,” “cell‐based meat,” “clean meat,” “artificial meat,” and “synthetic meat.” The production of cultured meat involves the division and differentiation of animal cells on a substrate. For this purpose, stem cells obtained from animals through biopsy not only have the ability to proliferate but also the potential to transform into different cell types, such as muscle and fat cells. These proliferating and differentiating cells come together to form cultured meat products (Fatima et al. 2023; Siddiqui et al. 2022).

The literature suggests that cultured meat can be seen as a sustainable alternative to traditional meat for many explanations, such as lower environmental impact (Tuomisto and Teixeira De Mattos 2011), being considered more ethical in terms of animal welfare (Rolland et al. 2020), production being more sustainable than traditional meat (Fu et al. 2023), and ability to achieve sensory quality characteristics close to traditional meat (Lee et al. 2024). Cultured meat is not yet at sufficient production level to be offered to consumers. In addition, some reasons are associated with the negative attitudes of consumers toward cultured meat. Studies show that reasons such as unnaturalness, food neophobia, unhealthiness, negative sensory expectations and high prices are associated with negative attitudes toward cultured meat (Chen et al. 2023; Rombach et al. 2022; Ruzgys and Pickering 2020; van Dijk et al. 2023).

This study aims to evaluate consumer attitudes toward cultured meat, which is not a part of a living animal but is produced in the laboratory using muscle stem cells, and to examine factors shaping consumer acceptance.

## 2 Method

### 2.1 Study Design

This cross sectional study was conducted between May 2024 and January 2025 on 504 young adults aged 18–40 years. A snowball sampling method used to recruit participants. Ethical permission was obtained to conduct the study, and participants were included in the study with informed consent. Scientific professionals educated in cultured meat and those without digital literacy were not included in the study.

### 2.2 Data Collection

The study data were collected through an online questionnaire form (Google Forms) and participants were invited to the study via phone and email. The questionnaire consists of eight sections: general information, health status, meat consumption habits, information on cultured meat, expectations and concerns toward cultured meat, sustainable and healthy eating behaviors (SHEB), food neophobia, and animal welfare.

In the first section of the questionnaire, general information about participants was collected, including age, sex, and educational status. In the second section, participants were asked about the presence of any medically diagnosed diseases. Additionally, self‐reported body weight (kg) and height (cm) were recorded. The third section included questions related to meat consumption, such as meat consumption frequency, reasons for consuming meat, reasons for limiting or avoiding meat consumption, and meat preferences. In the 4th section, firstly, the status of having knowledge about cultured meat was asked and then brief information about cultured meat was given below. This section continued with questions about cultured meat consumption and alternative protein source preferences.

Cultured meat is a meat product that is not part of a living animal but is produced in a laboratory using muscle stem cells. These stem cells are obtained from live animals without causing them any discomfort. Cultured meat is also known as artificial meat, clean meat, or sustainable meat. The goal is to create a product that resembles traditional meat in appearance, smell, texture, and taste. Currently, this product is not available on the market in Türkiye and cannot be found in supermarkets or butcher shops. However, research is being conducted to introduce it as a potential new method of meat production in the future.

The 5th section includes questions designed to determine expectations and concerns toward cultured meat. The questions asked in this section were created by adapting the questions in the studies conducted by Choudhary et al. (2023) and Weinrich et al. (2020). The questions in this section were evaluated with a five‐point Likert scale (1: strongly disagree, 5: strongly agree). The SHEB scale in the 6th section, the Food Neophobia Scale (FNS) in the 7th section, and the Animal Welfare Attitudes (AWA) scale in the 8th section were used.

### 2.3 Sustainable and Healthy Eating Behaviors

In 2019, Zakowska‐Biemans and colleagues developed the SHEB scale (Żakowska‐Biemans et al. 2019). The scale consists of 34 items and is scored on a seven‐point Likert scale. The Turkish validity and reliability study was conducted by Köksal et al. (2023) and was completed to have 32 items. The scale consists of seven factors in total, including quality label, seasonal food and avoiding food waste, animal welfare, meat reduction, healthy and balanced nutrition, local food and low fat. A seven‐point frequency scale ranging from (1) never through (4) sometimes to (7) always was used. Factor scores are calculated by taking the average of the points given to the items in that factor. When calculating the total scale score, the average of the points given to all factors is taken.

### 2.4 Food Neophobia Scale

Pliner and Hobden developed the FNS in 1992 as a psychometric instrument to detect food neophobia (Pliner and Hobden 1992). The Turkish validity and reliability study of the study was conducted by Duman et al. (2020). This scale consists of 10 items, five positive and five negative, related to food and food consumption. The FNS is evaluated with a seven‐point Likert scale (one: strongly disagree, seven: strongly agree). Negative items numbered 1, 4, 6, 9, and 10 on the scale are scored by reversing, resulting in a total scale score between 10 and 70 points.

### 2.5 Animal Welfare Attitudes

The AWA scale was developed by Randler et al. (2018). This scale provides a 20‐item scoring to determine attitudes of participants toward animal welfare. The scale consists of a five‐point Likert‐type response format and seven of the 20 items are scored by reversing. The total score is calculated by adding the responses to all items. A higher scale score reflects higher pro‐animal attitudes (Randler et al. 2018). The scale was used by translating the items through parallel translation (Randler, Ballouard, et al. 2021). In another study involving 22 countries, including Türkiye, the scale was used in Turkish and the reliability results for the scale were also included in this study (Randler, Adan, et al. 2021).

### 2.6 Statistical Analysis

The categorizable data of the study are expressed as number (*n*) and percentage (%), and numerical data are expressed as mean (X) and standard deviation (±SD). The normal distribution status of the data was determined using visual and analytical methods. In the statistical evaluation of the data, parametric or nonparametric tests were applied depending on whether the data showed normal distribution or not. Multiple linear regression analysis was applied to explore the factors affecting expectations and concerns toward cultured meat. Structural equation modeling was used to investigate the degree of association between cultured meat acceptance, expectations, concerns, sustainable nutrition, food neophobia, and animal welfare. To assess the model fit of the structural equation model, commonly used fit indices were applied, including the Chi‐square to degrees of freedom ratio (CMIN/DF), Comparative Fit Index (CFI), Root Mean Square Error of Approximation (RMSEA), and Tucker‐Lewis Index (TLI). Statistical significance level was accepted as *p* < 0.05. Statistical analyses were performed using AMOS and SPSS 26.0.

## 3 Results

The study included 504 participants (328 female and 176 male). Between the ages of 18 and 40, the mean age of participants was 24.4 ± 6.05. Most of the participants (81.3%) had a university degree or higher. Descriptive information about the participants is provided in Table 1.

**TABLE 1:** Descriptive characteristics of participants.

	*n*	%		*n*	%
**Sex**			**Working status**		
Female	328	65.1	Working	149	29.6
Male	176	34.9	Not working	355	70.4
**Educational status**			**Place of residence**		
Primary school	8	1.6	Urban	452	89.7
High school	86	17.1	Rural	52	10.3
University	366	72.6		**X**	**SD**
Postgraduate	44	8.7	Age (year)	24.4	6.05
**Presence of chronic disease**			Height (cm)	168.2	9.52
Yes	72	14.3	Body weight (kg)	67.9	16.06
No	432	85.7	BMI (kg/m^2^)	23.8	4.36

Abbreviation: BMI, body mass index.

Although the reasons for participants consuming meat are delicious (64.1%) and necessary for the body (57.5%), the high price (72.2%) has a high rate among the reasons for restricting/not consuming meat. 61.1% of the participants have heard of the concept of cultured meat before, and 33.5% stated that they could consume cultured meat. Among alternative protein sources, legumes were determined to be the most preferred (78.6%), followed by plant‐based meat and cultured meat with lower rates. Table 2 provides knowledge about meat consumption and cultured meat of participants.

**TABLE 2:** Participants knowledge about meat consumption and cultured meat.

	*n*	%		*n*	%
**Frequency of meat consumption**			**Meat preference**		
6–7 per week	24	4.8	Red meat	258	51.2
4–5 per week	88	17.5	Poultry (white meat)	222	44.0
2–3 per week	214	42.5	Seafood	22	4.4
1 per week	93	18.4	Plant‐based meat/vegan meat	2	0.4
1–3 per month	58	11.5	**Hearing of cultured meat**		
Less than once a month or never	27	5.3	Yes	308	61.1
**Reason for consuming meat** ^a^			No	196	38.9
Natural	83	16.5	**Knowing about cultured meat**		
Healthy	190	37.7	Yes	119	23.6
Delicious	323	64.1	Partially	192	38.1
Necessary for the body	290	57.5	No	193	38.3
Satisfying	130	25.8	**Intention to consume cultured meat**		
**Reason for restricting/not consuming meat** ^a^			Yes	169	33.5
High price	364	72.2	No	335	66.5
Dislike the taste	100	19.8	**Alternative protein source preference** ^a^		
Disgust	55	10.9	Legumes	396	78.6
Not suitable for animal welfare	20	4.0	Algae	19	3.8
Depletion of natural resources	31	6.2	Insects	9	1.8
Vegetarian/vegan diet	11	2.2	Plant‐based meat	56	11.1
			Cultured meat	30	6.0
			None	78	9.5

^a^ More than one option has been selected.

It is seen that the expectations of the participants regarding cultured meat are not positive (*t* = −9.649; *p* < 0.05), but the participants had high concerns (*t* = 15.468; *p* < 0.05). One‐sample *t*‐test results for both participant expectations and concerns regarding cultured meat are presented in Table 3.

**TABLE 3:** Expectations and concerns of participants regarding cultured meat (test value: 3).

	X	SD	T2B	*t*	*p*
**Expectations**					
I believe that cultured meat is healthy.	2.4	1.01	10.5%	−13.487	**< 0.001**
I believe that cultured meat is good for the environment.	2.7	1.14	24.4%	−6.356	**< 0.001**
Cultured meat will reduce the impact of global warming associated with agriculture.	2.8	1.12	28.2%	−3.822	**< 0.001**
I believe that cultured meat is beneficial for animal welfare and will reduce animal suffering.	2.9	1.27	35.9%	−2.657	**0.008**
Cultured meat is generally considered safe.	2.4	1.02	12.3%	−13.845	**< 0.001**
Cultured meat does not pose a serious health risk.	2.5	1.05	13.1%	−11.314	**< 0.001**
Cultured meat is not dangerous to human health.	2.5	1.01	12.3%	−12.223	**< 0.001**
All in all, cultured meat is good for future generations.	2.5	1.10	17.9%	−10.129	**< 0.001**
It is likely that cultured meat will replace traditional meat production.	2.7	1.17	28.6%	−5.643	**< 0.001**
Cultured meat can help solve global food shortages.	2.9	1.19	36.5%	−1.270	0.205
**Mean**	2.6	0.89		−9.649	**< 0.001**
**Concerns**					
Cultured meat is likely to have negative long‐term health effects.	3.5	1.17	54.8%	10.396	**< 0.001**
Cultured meat may have long‐term negative environmental impacts.	3.4	1.14	48.0%	8.391	**< 0.001**
Evidence that cultured meat production has a low carbon footprint is insufficient.	3.3	1.01	34.7%	5.684	**< 0.001**
The safety of cultured meat is not yet supported by sufficient scientific evidence and data.	3.5	1.05	48.1%	10.087	**< 0.001**
Cultured meat does not appear to be healthier than traditional meat and meat products.	3.7	1.17	60.9%	13.174	**< 0.001**
Cultured meat may pose a danger to future generations.	3.6	1.12	52.6%	11.422	**< 0.001**
I believe that cultured meat is not natural.	3.8	1.14	67.6%	16.502	**< 0.001**
Growing meat in a bioreactor seems to reduce the natural quality of meat and meat products.	3.7	1.07	60.9%	14.439	**< 0.001**
Rapid adoption of cultured meat and related technologies could be risky.	3.8	1.09	66.9%	16.508	**< 0.001**
Society should not rely primarily on cultured meat production to solve food‐related issues.	3.8	1.11	67.6%	16.846	**< 0.001**
**Mean**	3.6	0.89		15.468	**< 0.001**

*Note:* *p* values in bold indicate statistically significant results (*p* < 0.05).

The mean score of the participants from the SHEB scale was found to be 4.1 ± 1.06. The mean score on the FNS was 39.5 ± 8.21, whereas it was calculated as 69.0 ± 9.98 on the animal welfare scale. Scale scores are shown in detail in Table 4.

**TABLE 4:** SHEB, FNS and AWA scale scores of the participants.

	X	SD
**SHEB**		
*Total score*	4.1	1.06
Quality label	4.0	1.08
Seasonal food and avoiding food waste	4.4	1.18
Animal welfare	3.9	1.41
Meat reduction	3.6	1.33
Healthy and balanced nutrition	4.6	1.38
Local food	3.7	1.41
Low fat	4.3	1.37
**FNS**	39.5	8.21
**AWA**	69.0	9.98

Abbreviations: AWA, animal welfare attitudes; FNS, food neophobia scale; SHEB, sustainable and healthy eating behaviors.

Table 5 shows the multiple linear regression model used to predict the mean scores for expectations and concerns. A one‐unit increase in AWA score was associated with a 0.016‐unit (β = 0.016, SE = 0.004, 95% CI [0.008;0.024]) increase in the expectations score, whereas a one‐unit increase in educational status corresponded to a 0.146‐unit increase (β = 0.146, SE = 0.070, 95% CI [0.009;0.283]). In the regression model, where expectations were the dependent variable, one‐unit increases in age, SHEB score, and FNS score were significantly linked to decreases of 0.026 (β = −0.026, SE = 0.007, 95% CI [−0.039;‐0.013]), 0.206 (β = −0.206, SE = 0.035, 95% CI [−0.276;‐0.137]), and 0.009 units (β = −0.009, SE = 0.005, 95% CI [−0.018;‐0.001]) in the expectations score, respectively (*p* < 0.05). Additionally, a one‐unit increase in SHEB was associated with a 0.085‐unit (β = 0.085, SE = 0.037, 95% CI [0.012;0.159]) increase in the concerns score (*p* < 0.05).

**TABLE 5:** Multiple linear regression model for the prediction of expectations and concerns mean score.

Independent variables	Unit of variables	Expectations				Concerns			
		β	SE	Cl 95%	*p*	β	SE	Cl 95%	*p*
Age (year)	Number	−0.026	0.007	−0.039;‐0.013	**< 0.001**	0.014	0.007	−0.001;0.028	0.059
BMI (kg/m^2^)	Number	−0.001	0.009	−0.020;0.017	0.884	0.010	0.010	−0.009;0.030	0.294
SHEB	Number	−0.206	0.035	−0.276;‐0.137	**< 0.001**	0.085	0.037	0.012;0.159	**0.023**
FNS	Number	−0.009	0.005	−0.018;‐0.001	**0.038**	0.001	0.005	−0.009;0.010	0.903
AWA	Number	0.016	0.004	0.008;0.024	**< 0.001**	0.007	0.004	−0.002;0.015	0.113
Sex	Categorical 1: Male 2: Female	0.105	0.086	−0.063;0.274	0.220	0.086	0.091	−0.093;0.265	0.344
Educational status	Categorical 1: Primary school 2: High school 3: University 4: Postgraduate	0.146	0.070	0.009;0.283	**0.036**	0.019	0.074	−0.126;0.164	0.794
		Adjusted *R* ^2^: 0.139				Adjusted *R* ^2^: 0.022			

*Note:* *p* values in bold indicate statistically significant results (*p* < 0.05).

Abbreviations: AWA, animal welfare attitudes; BMI, body mass index; FNS, food neophobia scale; SHEB, sustainable and healthy eating behaviors.

**FIGURE 1:**
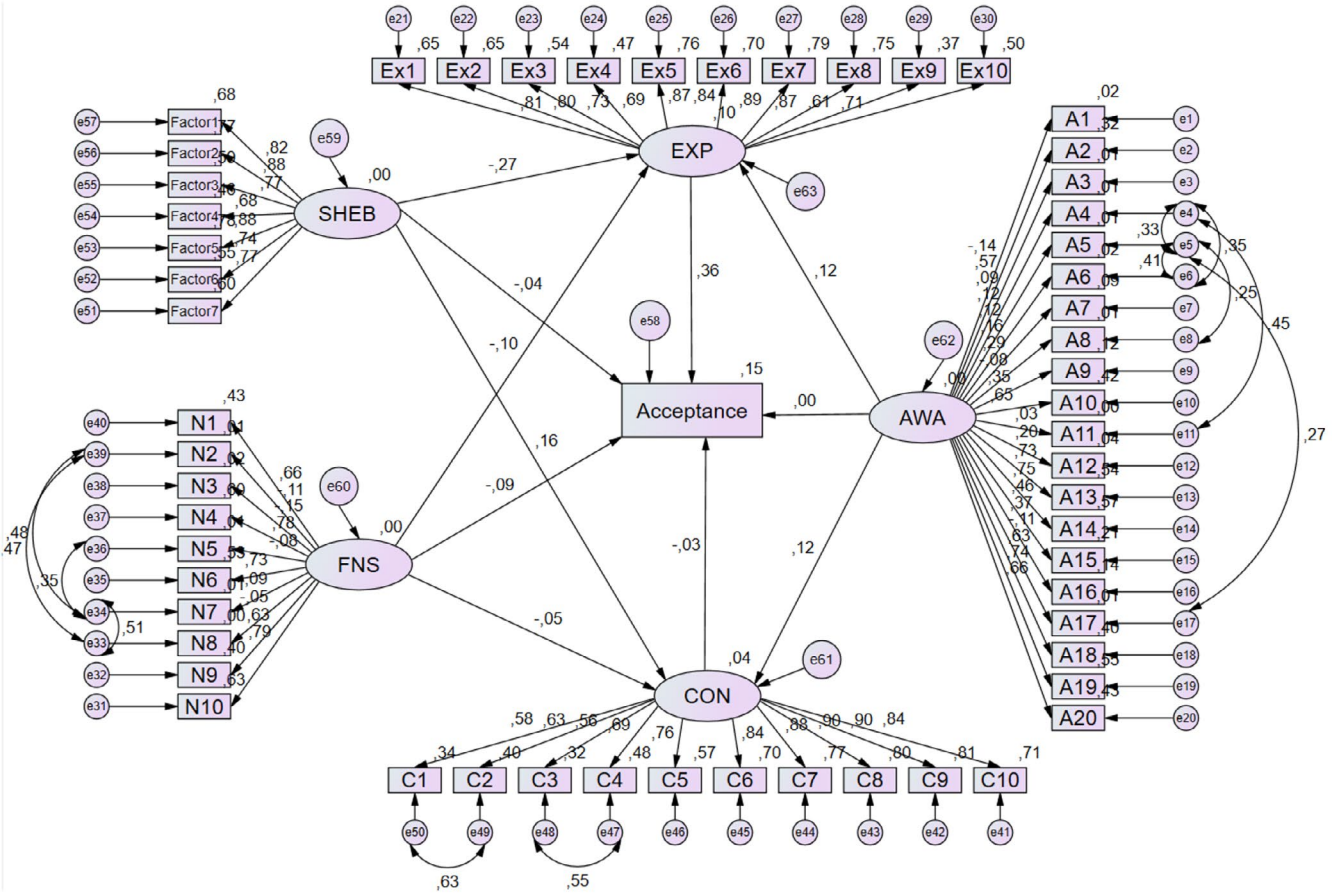
Structural equation models. CMIN/DF:3.009, CFI:0.811, RMSEA:0.063, TLI:0.802. AWA, animal welfare attitudes; CON, concerns; EXP, expectations; FNS, food neophobia scale; SHEB, sustainable and healthy eating behaviors.

The hypothesized relationships among SHEB, FNS, AWA, expectations, concerns, and acceptance of cultured meat are presented in the structural equation models diagram (Figure 1). The relationships between the variables are shown using arrows and boxes. The numbers above each arrow represent the standardized regression coefficients. The goodness of fit indices for the model indicate an acceptable fit (CMIN/DF:3.009, CFI:0.811, RMSEA:0.063, TLI:0.802).

According to the structural equation model estimation results presented in Table 6, SHEB negatively affect cultured meat expectations (β: −0.248; *p* < 0.001), whereas it positively affects cultured meat concerns (β: 0.169;< *p* < 0.001). Increasing attitudes toward animal welfare positively affect both cultured meat expectations (β: 0.137; *p* < 0.05) and concerns (β: 0.154; *p* < 0.05). Increasing food neophobia negatively affects cultured meat expectations (β: −0.060; *p* < 0.05), whereas it did not have a significant effect on cultured meat concerns. In addition, as expectations increase, acceptance of cultured meat increases (β: 0.207; *p* < 0.001), whereas concerns did not have a significant effect on acceptance of cultured meat.

**TABLE 6:** Results from the structural equation models of acceptance of cultured meat.

Path analysis	SE	CR	*p*	Standardized coefficient
SHEB → Expectations	0.043	−5.809	**< 0.001**	−0.248
SHEB → Concerns	0.049	3.432	**< 0.001**	0.169
AWA → Expectations	0.053	2.587	**0.010**	0.137
AWA → Concerns	0.063	2.464	**0.014**	0.154
FNS → Expectations	0.029	−2.057	**0.040**	−0.060
FNS → Concerns	0.034	−0.986	0.324	−0.034
SHEB → Acceptance	0.024	−0.892	0.373	−0.021
AWA → Acceptance	0.029	0.100	0.920	0.003
FNS → Acceptance	0.016	−1.954	0.051	−0.031
Expectations → Acceptance	0.027	7.720	**< 0.001**	0.207
Concerns → Acceptance	0.022	−0.764	0.445	−0.017

*Note:* *p* values in bold indicate statistically significant results (*p* < 0.05).

Abbreviations: AWA, animal welfare attitudes; FNS, food neophobia scale; SHEB, sustainable and healthy eating behaviors.

## 4 Discussion

The consumption of meat, one of the primary protein sources in diet, is influenced by various economic, cultural, ethical, and health‐related factors. However, the concept of sustainable nutrition encourages the reduction of meat consumption due to the environmental impacts of meat production such as greenhouse gas emissions, water consumption, and land use. In this context, alternatives such as legumes, algae, insects, plant‐based protein sources, and cultured meat are presented as substitutes for traditional meat consumption. This study aimed to evaluate the factors affecting consumer acceptance toward cultured meat. The fact that cultured meat is not yet available on the market means that consumers lack real experience. Nevertheless, understanding consumer expectations and concerns about cultured meat plays a crucial role in determining how it will be integrated into the future food system and ensuring acceptance of cultured meat.

A systematic review has identified taste, health effects, familiarity, food neophobia, disgust, and social norms as key determinants in consumer acceptance of alternative protein sources. Furthermore, it has been reported that the acceptance of alternative proteins is lower compared to traditional meat, with insect‐based proteins having the lowest acceptance rate, followed by cultured meat. In contrast, legumes and plant‐based proteins have the highest acceptance levels among alternative protein sources (Onwezen et al. 2021). Similarly, Gomez Luciano et al. found that participants showed the greatest interest in plant‐based protein sources among alternative proteins (Gómez‐Luciano et al. 2019). Consistently, in the present study, legumes exhibited the highest acceptance rate (78.6%), whereas plant‐based meat (11.1%) and cultured meat (6.0%) were observed to have lower acceptance levels (Table 2). The results of this study align with the general trends presented in the literature. Consumers tend to prefer more natural and familiar plant‐based protein sources. The low acceptance of cultured meat can be explained by factors, such as health concerns, taste, unnaturalness, food neophobia, and social norms.

Studies conducted in the United States have reported varying levels of acceptance toward cultured meat. In one study, 65.3% of participants responded positively to trying cultured meat (Wilks and Phillips 2017) while another study found that 66.4% were willing to try it, 48.9% were willing to consume it regularly, and 55.2% would prefer it over traditional meat (Bryant et al. 2019). Studies conducted in Germany have reported varying results regarding participants' willingness to try cultured meat (57%–70%) and their willingness to consume it regularly (30%–57%) (Jacobs et al. 2024; Weinrich et al. 2020). In Italy, 54% of participants expressed willingness to try cultured meat, whereas 44% considered purchasing it (Mancini and Antonioli 2019). Another study conducted in China, India, and the United States found that 59.3% of participants in China, 56.3% in India, and 29.8% in the United States were willing to purchase cultured meat (Bryant et al. 2019). In a cross‐country study conducted by Gómez‐Luciano et al. (2019), willingness to purchase cultured meat was observed at 20% in the United Kingdom, 42% in Spain, 11.5% in Brazil, and 15% in the Dominican Republic. In Africa, whereas 64% of participants had heard of cultured meat, only 8.9% were definitively willing to try it (Kombolo Ngah et al. 2023). In the present study, 61.1% of participants had heard of cultured meat, and 33.5% stated that they would be willing to consume it (Table 2). These results indicate that the acceptance of cultured meat varies significantly across countries and cultural structures. Notably, there is a significant difference between having information about cultured meat, trying it, and consuming it regularly. Therefore, variables, such as cultural norms, religious beliefs, and dietary habits should be considered to assess the global acceptance of cultured meat. Additionally, the inclusion of participants from different age groups in studies may also influence the acceptance of cultured meat. Younger individuals tend to be more open and willing to try cultured meat, whereas older age groups generally show more skepticism (Jacobs et al. 2024). These age‐related differences suggest that technological familiarity and risk perception may play an important role in shaping consumer attitudes toward cultured meat across populations.

Research on consumer expectations and concerns regarding cultured meat has yielded varying results. Wilks and Phillips reported that consumers perceive cultured meat as less natural, less attractive, and less delicious than traditional meat. However, they agreed that it is environmentally friendly and carries a lower risk of zoonotic diseases. Additionally, the idea that cultured meat could enhance animal welfare was widely accepted among consumers. Nevertheless, expectations regarding potential to address food shortages and mitigate environmental impacts were found to be relatively lower (Wilks and Phillips 2017). Baybars et al. (2023) reported that participants did not consider cultured meat to be ethical, natural, healthy, tasty, or safe, even if they would consider it a viable alternative to traditional meat in the future. Weinrich et al. (2020) stated that prior knowledge of cultured meat, along with ethical advantages such as contributions to animal welfare and environmentally friendly, serve as positive driving forces for the willingness to try and consume cultured meat. In contrast, regardless of prior knowledge, perceptions of unnaturalness and the belief that it is less flavourful were identified as negative driving forces influencing the willingness to try and consume cultured meat. Jacobs et al. (2024) found that the most important factors in the willingness to try cultured meat were ethics, curiosity, and environmental friendliness. In a study of meat scientists, the main reasons cited for the lack of acceptance of cultured meat were reliance on traditional meat, distrust of a new and untested technology, and high cost. However, most of participants recognized the positive environmental and animal welfare impacts of cultured meat (Choudhary et al. 2023). In the present study, expectations were primarily focused on the potential of cultured meat to improve animal welfare and address food shortages. However, concerns were notably high regarding various aspects such as unnaturalness, the risks of rapid adoption, nutritional quality, and health implications (Table 3). It is known that consumer acceptance is shaped not only by the content of the product but also by how it is defined. Terms with technical or negative connotations, such as “lab‐grown meat” or “artificial meat” can reduce acceptance rates by raising doubts about the naturalness of cultured meat. In contrast, expressions that can create more neutral or positive perceptions, such as “cell‐based meat” or “cultured meat” can affect consumer attitudes more positively. In this context, how cultured meat is presented to the public can be considered a linguistic factor that can significantly affect the level of acceptance. Although previous studies have highlighted unnaturalness and taste as primary concerns, this study identified strong, multidimensional concerns beyond just unnaturalness. These results suggest that participants are not only worried about taste and naturalness but also express greater concerns regarding the long‐term health effects and technological adaptation processes associated with cultured meat. Although there are not enough studies, it is thought that cultured meat may have potential risks and benefits in terms of health. In the evaluation made by Food and Agriculture Organization and World Health Organization (2023), many potential hazards were identified at each stage of the production process. It was evaluated as a potential risk that some of the additives and equipment used may cause allergic reactions or that molecules with unknown biological effects, such as hormones, may be added to the environment (FAO and WHO 2023). The absence of detoxification mechanisms (such as liver and kidney) naturally found in living animals in cultured meat indicates the risk of toxic accumulation. In addition, factors affecting the shelf life of the product, such as maturation and storage processes, have not been sufficiently investigated (Hocquette et al. 2025). Since there is no myoglobin in cultured muscle tissue, differences in nutritional value, such as low iron content come to mind (Fraeye et al. 2020). On the other hand, since the composition of the culture environment can be regulated by researchers in a controlled manner, it is thought that it can reduce the risk of pathogen contamination in terms of product safety. In addition, the risk of transmission of animal‐borne diseases can be reduced in cultured meat (Ching et al. 2022). Therefore, it is important to clarify the health benefits and potential risks of cultured meat through further scientific research and to share this information transparently with the public in order to increase consumer confidence.

The demographic factors on the acceptance of cultured meat are also an important variable. Studies have shown that males are more willing to eat cultured meat than females, that the desire to try cultured meat decreases as age increases, and that young adults with higher education levels are more willing to try cultured meat (Jacobs et al. 2024; Wilks and Phillips 2017). It has been observed that the willingness to try cultured meat has reached the highest level, especially among individuals with a doctorate degree (Jacobs et al. 2024). In addition, it has been stated that countries with higher income and education levels are more willing to try cultured meat (Kombolo Ngah et al. 2023). Mancini and Antonioli (2019) determined that the potential consumer profile of cultured meat consists of individuals who are young, highly educated, familiar with the concept of cultured meat, consume meat, and are willing to reduce meat consumption. In this study, expectations regarding acceptance of cultured meat showed a negative change with age and a positive change with education level similar to the literature. However, no significant relationship was found between sex and cultured meat expectations. In addition, none of these demographic factors showed a significant connection with concerns about cultured meat (Table 5). The acceptance of cultured meat as education level increases can be associated with participants with higher education levels being more open to food technologies and increasing sustainability awareness.

Consumer acceptance of cultured meat is shaped by individual attitudes and habits as well as environmental and ethical concerns. Awareness of sustainable nutrition increases the interest of individuals in food alternatives with low environmental impacts, whereas food neophobia may cause resistance to new and unfamiliar foods. On the other hand, sensitivity to animal welfare may encourage avoidance of traditional animal products due to ethical concerns. Fu et al. reported that food technology neophobia hindered the acceptance of cultured meat, but the perceived unnaturalness of cultured meat had no significant effect on acceptance. Furthermore, perceiving cultured meat as a sustainable alternative did not significantly increase purchase intention (Fu et al. 2023). Hwang et al. (2020) found food neophobia to be a statistically significant negative motivator among the factors influencing the purchase of cultured meat, whereas sustainability was not a significant positive motivator. Palmieri et al. (2021) stated that consumers who think that cultured meat can improve animal welfare and reduce environmental problems are more likely to accept cultured meat. Rehman et al. stated that sustainable consumption and animal welfare are important factors in the preferences of consumers for meat alternatives, but the healthiness of the product is the primary determinant. In addition, differences were observed between age groups. In older age groups, health and nutrition factors were more important, whereas in younger age groups (16–35 years), sustainable consumption and animal welfare were found to be more important (Rehman et al. 2024). In this study, it was observed that SHEB and sensitivity to animal welfare increased concerns about cultured meat, whereas food neophobia decreased expectations. However, all three factors did not directly affect the acceptance of cultured meat. On the other hand, expectations about cultured meat were found to be one of the strongest factors influencing acceptance, whereas concerns did not directly affect acceptance (Table 6). Consistent with the literature, food neophobia was found to indirectly negatively affect cultured meat acceptance by lowering expectations. Although previous studies have indicated that sustainability has a positive impact regardless of food neophobia, the present study found that SHEB increased concerns about cultured meat. This may be related to consumer knowledge about sustainable foods and perceptions of whether cultured meat is genuinely sustainable. The SHEB scale used in this study encompasses not only sustainability but also healthy eating, which has been identified as a significant concern associated with cultured meat acceptance in other research. The results on animal welfare suggest individual differences in perceptions of whether cultured meat provides an ethical solution. Consumers who prioritize animal welfare may experience greater concerns due to the unnatural nature of laboratory‐based production or development for commercial purposes, rather than viewing it as an ethical alternative. The results indicate that expectations regarding cultured meat are among the strongest predictors of acceptance, whereas concerns do not directly influence acceptance. This suggests that participants focus more on cultured meat expectations, whereas concerns do not play a decisive role in their consumption decisions. The discrepancies between studies suggest that consumer perceptions of cultured meat are multidimensional and contextually variable.

This study has several strengths and limitations. Given the limited research on the acceptance of cultured meat, this study provides an opportunity to examine the complex relationships among multiple variables using structural equation modeling. Incorporating diverse perspectives such as sustainable nutrition, animal welfare, and food neophobia broadens the scope of the study. However, the focus of this study on a specific demographic group (age, education status, or geographic region) may limit the direct generalization of the results to larger populations. Additionally, since participants had not previously tasted cultured meat, their expectations and concerns may not fully reflect actual consumption behaviors.

In conclusion, this study comprehensively addressed consumer expectations and concerns toward cultured meat, identifying the key factors affecting acceptance. Notably, sensitivity to sustainable nutrition and animal welfare was found to increase concerns about cultured meat but did not directly determine acceptance of cultured meat. In line with general trends reported in the literature, the results suggest that consumers tend to prefer plant‐based proteins, which they perceive as more natural and familiar, over cultured meat. Although acceptance of cultured meat varies across countries, consumer knowledge and experience with the product emerge as crucial factors that could influence acceptance rates. Furthermore, demographic analysis revealed that interest in cultured meat decreases with age, whereas acceptance rates increase with higher levels of education. The results of this study reveal that consumer expectations, especially those shaped around themes such as health, environmental sustainability, animal welfare, food safety, and sustainability, play a decisive role in the acceptability of cultured meat. In this context, it is of great importance to address consumer concerns regarding areas such as the safety, nutritional value, and environmental impact of cultured meat with transparent and scientific information. Researching whether cultured meat is a safe and nutritionally equivalent alternative to traditional meat will increase consumer confidence. However, the increasing diversity of plant‐based or other protein alternatives necessitates that cultured meat emphasizes unique contributions and complementary potential within this expanding ecosystem. As a result, the integration of cultured meat into sustainable food systems depends not only on technological advances but also on the effective alignment of product features with consumer expectations and societal priorities. Additionally, longitudinal studies examining how consumer attitudes change over time following the commercialization of cultured meat can provide important data on the sustainable acceptance of the product. In this context, different forms of communication such as informative labeling and visual representation of production details can contribute to product acceptance.

## Author Contributions

**Feray Gençer Bingöl:** data curation (equal), formal analysis (equal), investigation (equal), methodology (equal), visualization (equal), writing – original draft (equal). **Duygu Ağagündüz:** conceptualization (equal), data curation (equal), investigation (equal), methodology (equal), writing – review and editing (equal).

## Ethics Statement

This study was conducted according to the guidelines laid down in the Declaration of Helsinki, and all procedures involving research study participants were approved by the Burdur Mehmet Akif Ersoy University Non‐Interventional Clinical Research Ethics Committee. Written informed consent was obtained from all subjects.

## Conflicts of Interest

The authors declare no conflicts of interest.

## Data Availability

Data will be made available upon request.
